# Microscopy and Serological Assessment for Heartworm Infection in Cats in Makati, Philippines Showing Clinical Signs of Dirofilariosis

**Published:** 2013

**Authors:** A Baticados, W Baticados, G Coz, SMEAS Carlos, E Carlos

**Affiliations:** 1 Dept. of Veterinary Paraclinical Sciences, College of Veterinary Medicine, University of the Philippines Los Baños, Laguna 4031, Philippines; 2 Makati Dog and Cat Hospital (MDCH), 5426 General Luna St. cor. Algier St. Poblacion, Makati City, Metro Manila 1210, Philippines

**Keywords:** Antigen, Cats, *Dirofilaria immitis*, Microfilaria concentration method, Philippines

## Abstract

**Background:**

The sole published data on feline heartworm infection in the Philippines was reported four decades ago. The study therefore endeavoured to assess and provide an update on the current status of heartworm infection in domesticated feline species using serologic and parasitological examination methods.

**Methods:**

A total of 46 males and 54 females cats showing clinical signs of dirofilariosis from Makati City, Philippines were subjected to two antigen-based test kits and a microfilaria concentration method.

**Results:**

The most commonly observed clinical sign was coughing while exercise intolerance was seldom seen. Age groups ranging from 1 to 4 years old exhibited majority of the clinical signs whereas the 8.1 to 12 years category had the least. The results from the different detection methods employed revealed that none of the animals were positive for circulating microfilaria and no detectable levels of heartworm antigens were obtained.

**Conclusion:**

The presence of associated clinical signs is not an outright indicator of feline dirofilariosis and may be indicative of the rarity of heartworm infection in cats in Makati, Philippines.

## Introduction

*Dirofilaria immitis*, commonly known as canine heartworm is a mosquito-borne filarial nematode of veterinary and public health importance (1). The parasite is ubiquitously found in all continents (2) and has continued to exhibit its potential as a zoonotic disease since 1980's (3, 4). The parasite can occasionally infect man (5, 6) but is unable to complete maturation in humans and instead dies lodged within the lungs forming an embolus or a pathologic cyst-like lesion (5, 7). Although man is considered a dead-end host, several cases of human dirofilariasis involving different organ systems and stages, including adult forms have been reported.

There are several cases in humans which involve immature stages of the parasite. Young parasite stages were ectopically found in the liver (8); lodged in the spermatic cord (9); eye (10); right side of the heart (11) and most commonly in the pulmonary area (6, 12, 13). Interestingly, a case involving two adult female worms of *Dirofilaria immitis* from the heart and inferior vena cava were obtained from a 36-year-old Japanese man who died from liver cirrhosis (14). Canine species is the prime reservoir host for human dirofilariasis. However, the potential of feline species as a source of human infection cannot be discounted since *D. immitis* infection is now also considered as a potential causative agent of heartworm in feline species (15, 16). Accordingly, Myszak (17) stated that presence of heartworm in canines increases the likelihood for feline heartworm infections as well. Moreover, reports of increasing numbers of feline heartworm infections in other countries were documented (18). On the other hand, the sole report of heartworm infection in feline species in the Philippines was published late 1965, more than four decades ago (19).

Several factors contribute to the elusiveness of feline heartworm cases in the country. The primary reason is that in general cats are more resistant to adult *D. immitis* infection as compared with canines and usually very few L5 are able to reach the lungs 3 to 4 months after infection (20). Secondly, feline species exhibits nonspecific clinical signs, typically low worm burdens and a unique feline pathophysiology, which complicates its diagnosis. Thirdly, currently available heartworm tests, including antigen-based tests, have known limitations that make the diagnosis of FHD difficult (21).

Since the last known report of feline dirofilariasis was approximately forty years ago, the study therefore aimed to determine the current status of heartworm infection in cats from Makati, Philippines using three different diagnostic tests namely acetone microfilaria concentration method (Ohishi test), DiroCHEK ^®^ antigen test (Synbiotics Corporation, San Diego, California) and WITNESS^®^ HW antigen test (Synbiotics Corporation, San Diego, California) (22, 23).

## Materials and Methods

### Study animals

A total of 46 male and 54 female cats, not less than a year of age (Range: 1-17 years old) based on veterinary hospital records, from Makati, Philippines with signs of heartworm infection and no history of dirofilariasis medication were obtained via purposive sampling. Specifically, clinical signs identified to be associated with feline heartworm disease included coughing, dyspnoea, vomiting, diarrhoea, exercise intolerance, anorexia and weight loss. Furthermore, data on the patient's signalment (sex and age) and living environment (indoor or outdoor) were also determined.

### Blood collection

Approximately 2 ml of blood were collected from each sample animal via cephalic venipuncture. Blood was allocated into one millilitre for Ohishi's concentration technique and the remaining blood sample was allowed to stand. Afterwards, each serum was harvested and subjected to testing for the presence of *D. immitis* antigen. The procedure was duly approved by University of the Philippines Los Baños, College of Veterinary Medicine Institutional Animal Care and Use Committee (IACUC), Protocol No. 2010-16.

### *Acetone* microfilaria concentration method

The test was performed as previously described (24).

### Heartworm antigen test kits

The biological samples were processed and tested for *D. immitis* using two heartworm antigen commercial test kits, DiroCHEK^®^ (Synbiotics Corporation, San Diego, California) and WITNESS^®^ HW (Synbiotics Corporation, San Diego, California), according to the manufacturers’ instructions. DiroCHEK^®^ and WITNESS^®^ HW heartworm test kits are interpreted based on color reactions.

DiroCHEK^®^ multi-unit enzyme-linked immunosorbent assay (ELISA) makes use of microwells coated with antibodies directed against *D. immitis* antigen and horseradish peroxidase (HRP) antibody conjugate. On the other hand, WITNESS^®^ HW is a single unit antigen test based on rapid immunomigration (RIM) technology for detection of heartworm antigen in canine or feline blood. The method utilizes gold-labeled antibody conjugate and the formed antigen/antibody gold complex eventually migrates across a nitrocellulose membrane and finally reacts with a second antibody at the level of the test line.

## Results

The two antigen detection methods and acetone microfilaria concentration test employed revealed that none of the cats were positive for circulating microfilaria and no detectable levels of heartworm antigens were obtained although signs commonly associated with heartworm infection were observed.

Table 1 illustrates that more male cats displayed dyspnea (47.83%), vomiting (65.22%), diarrhoea (54.35%) and exercise intolerance (17.39%). On the other hand, a greater number of the female cats showed signs of coughing (88.88%) as well as anorexia (51.85%). Respiratory signs such as coughing were mainly observed in 1-4 years old and seldom in 12.1-17 years old felines. In the case of gastrointestinal disturbances, majority of the cats 1-4 years were diarrheic (60.38%) as well as vomiting (62.26%) whereas these signs were least observed in 8.1-12 years old cats at 40% prevalence. Relative to the housing type, the data showed that a greater number of animals living indoor manifested dyspnea (44.4%), vomiting (62.22%), diarrhoea (62.22%), anorexia (53.33%), exercise intolerance (15.56%) and coughing (82.22%) as compared to those raised outdoors.

**Table 1:** Frequency distribution of observed clinical signs in relation to sex, age and housing type

Parameter	Cough		Dyspnea		Vomiting		Diarrhoea		Anorexia		Exercise Intolerance	
	n	%	n	%	n	%	N	%	n	%	n	%
**Sex**												
Male (46)	33	71.74	22	47.83	30	65.22	25	54.35	22	47.83	8	17.39
Female (54)	48	88.88	22	40.74	24	44.44	29	53.70	28	51.85	7	15.56
Total	81		44		54		54		50		15	
**Age**												
1 to 4 (53)	50	92.45	27	50.94	33	62.26	32	60.38	27	50.94	6	11.32
4.1- 8 (29)	17	68.97	10	34.48	14	48.28	16	51.72	20	68.97	5	17.24
8.1- 12 (13)	11	84.62	4	36.36	5	38.46	4	30.77	2	15.38	3	23.08
12.1- 17 (5)	3	60.00	3	60.00	2	40.00	2	40.00	1	20.00	1	20.00
Total	81		44		54		54		50		15	
**Type of housing**												
Indoor (45)	37	82.22	20	44.4	28	62.22	28	62.22	24	53.33	7	15.56
Outdoor (55)	44	80.00	24	43.64	26	47.27	26	47.27	26	47.27	8	14.55

## Discussion

Although negative results were derived from the study, the possible presence of dirofilariasis in cats in the Philippines should not be entirely discounted because the parasite's existence was confirmed approximately four decades ago. As such, the clinical signs related to heartworm infection and its effect on the study parameters are discussed with the prospect of serving as a guide to further studies.

Majority of the clinical signs were seen in the 1-4 age group with coughing (92.45%) as the prominent clinical manifestation. A previous study in south-eastern Michigan states that cats <2 years of age have lesser chances of microfilariae infection than older cats (25). On the contrary, another study illustrated that the age group of positive (7.3 ± 5.1 years) and negative (6.6 ± 4.9 years) cats were not significantly different (18). These findings suggest that age is not a likely predisposing factor to determine heartworm infection in cats.

Both male and female cats showed variable clinical signs with coughing as the most prevalent finding. Furthermore, most of the clinical findings were observed in male cats. On a side note, a study by Atkins et al. (18) demonstrated that male feline species (86%) were more likely to come out positive than the female species (66%). In addition, data from a previous research indicated that non-domestic cats are at risk for heartworm exposure and infection and also male cats being at greater risk of exposure to the parasite infection (18).

In reference to the type of housing, although more outdoor cats represented the study population, there was a preponderance of related clinical signs to dirofilariasis infection in indoor cats i.e. those situated indoors were more prone to clinical manifestations. The studies of Miller et al. (26) and Atkins et al. (18), revealed that there were simultaneous increases in the risk of parasite infection in cats housed outdoors. However, these studies likewise indicated that indoor housing does not guarantee protection against *D. immitis* infection. Thus, indoor housing does not confer complete protection against *D. immitis* infection in cats (20, 21, 25, 27). Kalkstein et al. (25) further stated that since the type of housing of feline was shown not associated with *D. immitis* infection by several studies, cats should be considered candidates for heartworm prophylaxis regardless whether they are raised indoors or outdoors, as long as they are located in areas at risk of heartworm infection.

Table 1 shows coughing as the most frequently observed clinical sign in the sample population, while the least observed was exercise intolerance. The study of Atkins et al. (27) likewise showed that coughing was one of the strongest indicators of heartworm infection in cats. Conversely, Robertson-Plouch et al. (21) stated that although coughing, dyspnea and vomiting are associated with feline heartworm disease (FHD) these clinical signs may be indicators of conditions such as bronchitis, asthma, lungworm infection and other feline respiratory diseases as well. Similarly, Dhupa et. al. (28) indicated that a disease such as feline bronchial asthma is sometimes mistaken as heartworm disease. Furthermore, viruses like feline calicivirus (FCV) and feline herpesvirus-1 (FHV-1) are also commonly associated with feline respiratory disease especially in animals living together in large numbers such as pet stores, catteries and shelters (29). Correspondingly, since clinical signs associated with heartworm infection were observed, the animals were subjected to serological and parasitological tests.

The results from the different antigen-based detection methods (DiroCHEK^®^ and WITNESS^®^ HW) employed in the study revealed that none of the animals had detectable levels of heartworm antigens. DiroCHEK^®^ and WITNESS^®^ HW are commercially available heartworm test kits in the country that are interpreted based on color reactions. In addition, the antigen tests were designed to determine the presence of the antigen expressed by *D. immitis*; Aspartyl Protease Inhibitor Homologue (PDi33 antigen) which is reported to be present in all heartworm stages of the mammalian host (MCF, L3, L4, adult male and female) and primarily released by mature stages of heartworms in vitro (22). Antigen testing is not an absolute test and also has limitations. Although DiroCHEK^®^ antigen test is highly specific (98%) for *D. immitis* detection, its diagnostic sensitivity in cats is only 79% and this implies that reports may have been under-estimated and parasite prevalence in the field might be higher than reported earlier (25). Furthermore, since biologically cats can only harbor very few numbers of worms, these results to a parallel low production of heartworm antigen and below detectable levels of antibodies (30). Consequently, the success in the use of antigen tests is dependent on the amount of antigen released by mature adult female heartworms (31). These tests are very highly specific and will detect antigens exclusively from female heartworms that are at least seven or eight months old but do not generally detect infections that are less than five months old (7). However, it was also exhibited that although the antigen test is a valuable adjunct to *D. immitis* infection diagnosis, the test was observed to be less sensitive and prone to false negatives (32).

Subsequently, the acetone microfilaria concentration method likewise ascertained that all of the test samples did not possess circulating microfilaria. The use of microfilaria concentration technique in the demonstration of microfilariae (MCF) in the circulating blood though diagnostic has also its limitations. The demonstration of MCF is rendered inapposite whenever animal infections are less than six months in duration (20). This test will only detect infections with at least one sexually mature male and female heartworm species and single-sex infections are not diagnosed by this method. The occurrence of immune-mediated occult infections is also common (33, 34) and the test was reported to have poor sensitivity in detecting the presence of heartworms in clinical samples as well. Additionally, it was previously reported that the sensitivity of heartworm screening using microfilaria concentration techniques is around 20% more than that of direct smear method (35). The other reasons for false negative results in using microfilaria concentration techniques also include inadequate sample size and the host's immunity prior to therapy (24). Furthermore, it was believed that cats are probably imperfect hosts for *D. immitis*. The presence of circulating microfilaria in infected cats is rarely demonstrated, which usually persist approximately 195-228 days post infection. This is likely due to the known capability of cats to undergo host immune-mediated clearance of the microfilaria or probably reversible suppression of microfilaria production. This could explain the considerably shorter life span (2-3 years) of the parasite in feline species (20).

Overall, results of the study demonstrated that the presence of associated clinical signs did not result to positive observance of the parasite thus suggesting that the signs were non-specific and were not outright indicators of feline dirofilariasis in Makati City, Philippines.

## Conclusion

The different detection methods employed in the study were not able to reveal circulating microfilaria and detectable levels of heartworm antigens from the feline blood samples. The negative results of the study seem to agree with the previous report, which advocated that antigen tests are not paramount for the detection of FHD (25). Consequently, the study established that the presence of associated clinical signs is not an outright indicator of feline dirofilariasis. It is more likely that the clinical signs are non-specific in nature. Overall, the results derived from the study are analogous to previous reports (4, 13, 30) that specified that heartworm infection in feline species are rarely if at all detected. Further studies on a larger scale, scope and demography are advocated in combination with genomic testing to reliably diagnose or rule out *D. immitis* infection.
